# AllianceBlockchain in the Governance Innovation of Internet Hospitals

**DOI:** 10.3390/s25010142

**Published:** 2024-12-29

**Authors:** Xiaofeng Wang, Xiaoguang Yue, Ahthasham Sajid, Noshina Tariq

**Affiliations:** 1 College of Management, Shenzhen University, Shenzhen 518060, China; 2 Multimedia University, Cyberjaya 63100, Malaysia; ahthasham.sajid@riphah.edu.pk; 3 Department of Information Security and Data Science, Riphah Institute of Systems Engineering, Riphah International University, Islamabad 46000, Pakistan; 4 Department of Artificial Intelligence and Data Science, National University of Computer and Emerging Sciences, Islamabad 44000, Pakistan; noshina.tariq@isb.nu.edu.pk

**Keywords:** blockchain, electronic health records, Internet hospitals, distributed role-based access control, Rivest–Shamir–Adleman (RSA), security

## Abstract

The rise of Internet hospitals has significant issues associated with data security and governance in managing sensitive patient data. This paper discusses an alliance blockchain (i.e., a private blockchain) model for governance innovation in internet hospitals with an improved encryption methodology. We compare our proposed model, improved Rivest–Shamir–Adleman (RSA) encryption, integrated into the blockchain framework. Improved RSA achieves impressive improvements in all key metrics by increasing the throughput by 24.7% and lowering the latency by 19.8% compared to the base model. Thus, the improved model is more optimized for processing transactions related to healthcare data. Memory usage was also reduced by 14.3%. While encryption time remained pretty close, the decryption time remarkably improved by 97.5%. IoT sensors are one of the foundations for Internet hospitals that produce consistent patient data streams, such as physiological and environmental metrics. The proposed alliance blockchain model enables the secure and efficient real-time management of this sensor data. These results demonstrate the capability of alliance blockchain and cryptographic upgrades in creating safe and efficient governance frameworks for Internet hospitals.

## 1. Introduction

Rapid technological advances in healthcare have given birth to new service models, such as Internet hospitals [1,2]. Internet hospitals provide patients with healthcare services at a distance through virtual consultation, diagnosis, treatment, and follow-up without any face-to-face consultation. It is precious in extending services to rural and underserved populations, reducing costs, and improving access to medical expertise. However, with all these facilitations from digital health come significant challenges, the central area of which is data confidentiality or privacy [3,4]. Most information is susceptible to patient detail and, in a strict view, regulated as required by the Health Insurance Portability and Accountability Act (HIPAA) [5,6]. For this reason, online or Internet hospitals face substantial challenges regarding privacy and the integrity of governance regarding sensitive patient data over decentralized web space [7,8].

Blockchain technology has led in managing digital data in the finance, supply chain, and health industries concerning security and transparency [9,10,11]. For example, using a decentralized ledger, blockchain will provide multiple parties with shared, verifiable, and secure methods of storing data not exclusively dependent on a central authority. With health records, blockchain will improve data integrity with efficient access and interoperability of disparate systems [12,13]. Alliance blockchain is particularly well suited to healthcare environments as it is a semi-decentralized method where only a consortium of approved entities can participate in the network. Alliance blockchains balance the requirement for decentralization with regulatory and access control requirements unique to healthcare [14,15]. Alliance blockchain can offer Internet hospitals a secure, auditable, and collaborative platform for managing patient data across hospitals, clinics, and other healthcare providers.

However, the challenge in using blockchain in Internet hospitals is different. Internet hospitals have to manage a massive volume of transactions, such as patient data input, exchange of medical records, and real-time updates from the various IoT devices [16,17,18]. IoT sensors are a critical component of Internet hospitals because they create continuous data streams from wearable health monitors and environmental sensors, all to support real-time patient monitoring and care. Classic blockchain implementations induce latency and scalability issues caused by the computational complexity of consensus mechanisms and encryption protocols. More than that, existing encryption standards such as Rivest–Shamir–Adleman (RSA), although strong, may not be the best for blockchain transactions where speed and resource efficiency are crucial [19,20,21]. The trade-off between security and efficiency is more pronounced in healthcare, where data security cannot be compromised but must also meet the demands of real-time access.

The selection of RSA encryption is critical because it has been widely adopted in most public-key encryption algorithms because of its strong immunity to attacks, thereby ensuring the safe protection of sensitive data [22,23]. However, in the blockchain paradigm, the RSA performance tends to be inefficient due to the intensive computational operations about the generation, encryption, and decryption operations [24,25]. Thus, this paper presents an advanced governance model of Internet hospitals called alliance blockchain integrated with a novel RSA encryption algorithm named improved RSA. This improved RSA reduces the modulus computation of the initial RSA, so decryption time is reduced, latency is minimized, and throughput is improved without reducing the security aspect desired in managing healthcare information. This advancement is particularly crucial in a healthcare blockchain environment because high transaction throughput and low latency are needed to meet the real-time demands of both medical practitioners and patients.

This study evaluates the proposed system through a comparison with the state of the art. It measures the performance using key parameters relevant to healthcare applications: average encryption/decryption times, transaction latency, throughput, and memory usage. We thus concentrate on these parameters to highlight the feasibility and advantages of alliance blockchain with advanced encryption for Internet hospitals. It is a private blockchain model, ensuring controlled access by authorized entities. There are three contributions to this study. We propose an innovative Internet hospital governance system with alliance blockchain and an optimized RSA encryption model, ensuring safety and efficiency. Finally, we come up with the improved RSA model specifically designed to overcome the weaknesses of the traditional RSA model in blockchain environments and sharply improve decryption speed and resource efficiency. We conduct an elaborate examination of the designed model using critical performance metrics. Our solution will, therefore, establish itself as capable of providing what is necessary for the particular case of Internet hospitals. It addresses blockchain-specific challenges and, in addition, implements mechanisms to securely handle the sensor data coming from IoT, making it possible to integrate the sensor networks into the workflows of the Internet hospital efficiently while preserving privacy.

This work addresses the significant concern that Internet hospitals urgently require secure and efficient governance mechanisms. To that end, we used an alliance blockchain and developed an advanced encryption model in this paper. This paper contributes as follows:

1. A new governance framework for the Internet hospital based on a private alliance blockchain to safely and collaboratively manage healthcare data. It incorporates IoT sensors as primary data sources, allowing for the secure capture and processing of real-time sensor readings, such as patient vitals and environmental metrics.
2. It presents the improved version of RSA, specially designed for blockchain environments that support fast decryption and overall throughput. The advanced encryption method ensures optimal and privacy-preserving handling of high-frequency sensor data streams originated by healthcare IoT devices.
3. A comparison of both the base and improved RSA model regarding encryption time/decryption time latency, throughput, and memory usage. Performance evaluation shows the feasibility of the proposed model to process and secure the sensor-generated data within an Internet hospital while meeting scalability and real-time constraints.

The rest of this paper is divided into the following: Section 2 includes an overview of related works with blockchain applications to healthcare and optimization techniques for encryption. Section 3 and Section 4 outline the proposed architecture and methodology for the design of the alliance blockchain and how it implements the improved RSA model. Experimentation setup, results, and discussion for the evaluation are presented in Section 5. Lastly, Section 6 provides a conclusion of the paper.

## 2. Related Work

Blockchain technology has emerged as a transformative solution for managing and protecting sensitive data. For instance, healthcare electronic health records demand robust protection from unauthorized access and malicious attacks. Several studies have made immense contributions toward developing blockchain-based healthcare models that are secure, accessible, and efficient.

Zheng et al. [26] proposed a blockchain model for EHRs. They used digital signatures and used distributed ledger for transaction keeping. However, there was a trade-off between computational cost and processing time; therefore, it is inefficient for the real-time healthcare environment. Attribute-based encryption has been widely applied to improve access control and provide security for data-sharing healthcare applications. Therefore, Okamoto and Takashima [27] proposed an ABE scheme that involved non-monotone predicates, where the flexibility of access control is enhanced using a blockchain framework. However, this model needs the verification of attributes and may produce a higher computation overhead. Cao et al. [28] used a method of multi-authority attribute-based signature. This resolves the problems of forgery and collusion since the distributed authority is considered, and an attribute privacy can be utilized.

In addition, Das et al. [29] proposed a federated health alliance model for the next-generation IoT networks focused on secure data sharing between healthcare entities using blockchain. However, the introduced high latency due to the complex encryption processes negatively affects applications requiring real-time healthcare decisions. Misra et al. [30] discussed the integration of IPFS into healthcare blockchain models to store and retrieve medical records efficiently. However, IPFS-based systems have limitations, such as poor scalability and lack of real-time access in high-frequency transaction environments. To overcome these challenges, Srivastava and Gupta [31] introduced a modified logarithmic-based RSA algorithm integrated with SHA-256 in a blockchain framework to enhance security and computational efficiency. Li and Zhang [32] also proposed an attribute-based encryption based on blockchain technology to ensure efficient and secure data sharing in healthcare.

Despite the broad deployment of blockchain technology in smart healthcare, there remains a need for more literature on safely processing medical data in collaboration with CSP. For instance, Pirtle et al. [33] discussed integrating blockchain technology into the healthcare sector for social insurance to secure medical records and interact with insurance systems. Similarly, Hema and Márquez [34] proposed a system for peer-to-peer files using the InterPlanetary File System for securing and transferring patient health records. Scriber [35] presented a review-based work on blockchain, documenting its implementation in 23 projects across laboratories in cables. They used blockchain to authenticate medical data stored on cloud servers. Esposito et al. [36] proposed a blockchain architecture for processing healthcare data on cloud servers. Another comprehensive strategy for a blockchain-based CSP smart healthcare system proposed a blockchain framework protecting the operations of medical data on cloud servers in [37]. Chen et al. [38] utilized AI to support decisions related to disease detection and introduced a blockchain approach for the safe storage and sharing of medical data in CSP.

Furthermore, a blockchain-layered architecture was designed with IPFS storage integrated by Sanober and Anwar [39]. This used DNA encoding with modified AES and RSA with SHA-256 for encoding while transmitting and hashing. It also proposed reputation-based filtering attack detection and mitigation; however, it offers higher computational overhead due to complex encryption techniques. Khanh et al. [40] used RSA-encrypted NFTs and smart contracts to manage pediatric health records in their proposed blockchain-based framework. However, it involves increased complexity in RSA and NFT-based record management. Similarly, Trung et al. [41] proposed a blockchain-based framework using RSA-encrypted NFTs and smart contracts to increase pet health records’ security and transparent management. However, there is added complexity for implementing NFT-based data management. Sanober and Anwar [42] proposed a blockchain-layered architecture integrated with IPFS storage for secure healthcare applications. They used multilayer security mechanisms, such as DNA encoding, modified AES, RSA, and SHA-256, for encryption, transmission, and hashing. The model incorporates a reputation-based filtering mechanism that detects and prevents attacks. However, the proposed architecture incurs computational overhead because of the complexity of the encryption techniques.

In a nutshell, recent breakthroughs related to blockchain and encryption in healthcare have focused on enhancing data privacy, data integrity, and computational efficiency for processing. However, they suffer from security and performance trade-offs, especially in healthcare environments. Our study extends the works from earlier, incorporating an optimally optimized RSA-based encryption in an alliance blockchain for better security and efficiency toward real-time healthcare data management. It advances the critical idea that was first proposed with the goal of blockchain-enabled healthcare systems, which is to provide efficient transaction treatment and strong encryption to meet the requirements of the applications of Internet hospitals at any time. These research works formed the basis of this current work, a model for alliance blockchain proposing an RSA encryption optimization scheme. Further, higher transaction throughput, lower latency, and memory usage optimizations are being targeted. Accommodating cryptographic updates in the decentralized, multi-authority setting will enable the evasion of such limitations by providing an EHR management application that will ensure scalability. Table 1 summarizes and provides a quick comparison among state-of-the-art.

## 3. Proposed System Architecture

The proposed system architecture, based on private blockchain, provides the secure storage and management of medical data for patients in a decentralized environment. The design focuses on confidentiality, authenticity, and accessibility of data to improve security and reliability for the exchange of medical data in an Internet hospital. Below is the detailed framework of the improved model, building upon the base methodology with some significant optimizations.

1. Blockchain Infrastructure and Decentralization: The architecture is based on a distributed blockchain network that can be termed a decentralized database for EHRs. The blockchain framework supports nodes; each node represents an authority, such as a hospital, research center, or treatment facility. These authorities enroll patients, verify the data, and share them. Since it is designed in a distributed architecture, it eliminates the dependency on a single point of failure and enhances resistance to malicious attacks. IoT sensors as input data ensure continuous, real-time updates to the blockchain by monitoring comprehensive patient conditions through wearable devices and other sensors.
2. Improved RSA-Based Encryption: To ensure the integrity and security of the data, this paper incorporates an improved RSA algorithm in the blockchain. The improved algorithm utilizes optimized modulus computation that increases encryption strength but reduces the overhead compared to the base RSA. The improved RSA model also introduces a dynamic key generation process through a scaling factor to the logarithmic modulus to increase complexity in any unauthorized decryption attempt. The encryption approach to ensure secure access to high-frequency data inputs from IoT sensors will address the privacy issue associated with real-time sensor data.
3. Data Signing and Verification Mechanism: Every block in the blockchain uses a digital signature created by the improved RSA. This signature authenticates that the data has not been changed and originates from a validated source. Only parties with the right keys are allowed to generate or verify the signatures, which are verified on every transaction. This architecture provides a tamper-resistant mechanism, ensuring integrity from the start to the end of the data lifecycle. The sensor-generated data will be integrated into the system to ensure the secure and verified transmission of real-time readings to healthcare providers.
4. Distributed Role-Based Blockchain Encryption (DRB-BE): The proposed model embeds the Distributed Role-Based Blockchain Encryption (DRB-BE) scheme. It maps attributes of individuals like patients or doctors on their roles and permissions. Every authority in this system shall be able to properly and independently manage their key during encryption. Access controls enable the user to share data confidentially and in a manner that is even more relevant to their needs and policies. This architecture gives better privacy, ensuring unauthorized medical personnel do not view the secured clinical data. In this way, confidentiality regarding the patients is maintained. This model extends access control to include data collected from IoT sensors to ensure only authorized persons can view specific patient information derived from sensors.
5. Patient-Held Data Access: Patients will be given control over their medical data. They will also decide upon their desired permissions and can assign those across various entities in the Internet hospital network. This will serve the principles of data sovereignty, where patient authority will be enhanced regarding their health information with controlled security access through blockchain. Adding IoT sensors enables patients to monitor their real-time health data securely and selectively share, thereby encouraging greater engagement with their health.
6. Transaction Processing and Storage on the Blockchain: All data access, modification, and new data entry transactions are recorded separately as blocks in the blockchain. A block contains an identifier, timestamp, previous block hash, and the transaction details encrypted with improved RSA. The structure of the block ensures immutability and traceability. Hence, healthcare providers can audit all the transactions and maintain a transparent record of data interaction. This includes securing sensor data, ensuring real-time updates, and maintaining consistent blockchain records for healthcare applications.
7. Data Aggregation and Parameter Analysis: The system integrates modules for aggregating data and analyzing parameters to track and analyze patient data that crosses different authorities. These modules continuously check aggregated data based on throughput, latency, and memory usage, among other things. This analytical ability means the Internet hospital network makes sound decisions based on available information, optimizes resources, and ensures efficiency across the system. Sensor data from IoT devices are aggregated with other healthcare information, allowing in-depth analysis to make decisions and optimize patient care.

### Features of the Improved Model

The major features of the improved model are as follows:

1. Improved RSA: This is an improved version of the RSA encryption algorithm. It has a higher level of security than the altered model’s modified RSA and works more efficiently. Hence, it enhances the defense against attacks on cryptography. The improved RSA preserves data communicated from IoT sensors in Internet hospitals, integrating both high security and computational efficiency.
2. Decentralized Multi-Authority Access Control: The scheme proposed in the model is Multi-Authority Attribute-Based Encryption (MA-ABE) which supports decentralized, role-based access control; hence, many healthcare entities can share information without violating patient confidentiality. It includes protection of access to sensitive health metrics derived from the sensor.
3. Patient-Centric Data Management: The new model empowers patients with control over defining their permissions for access and allows them to share their information safely. It also follows the principles of health data sovereignty, as patients can control their sensor data sharing in real time.
4. Efficient Blockchain Transactions: The new model increases transaction throughput, reduces latency, and optimizes memory usage, thus making a more efficient and scalable blockchain-based EHR management system for large-scale deployment in Internet hospitals. This efficiency ensures that sensor data is managed with no losses in real time.
5. Immutability: Using the improved RSA encryption with the inherent properties of blockchain immutability has afforded considerable protection against unauthorized access. It also safeguards against tampering and cryptographic attacks, making the system robust and secure for handling healthcare data. The associated immutability features further benefit the inclusion of IoT sensor data, creating secure and traceable data records.

## 4. Proposed Methodology

**Figure 1:**
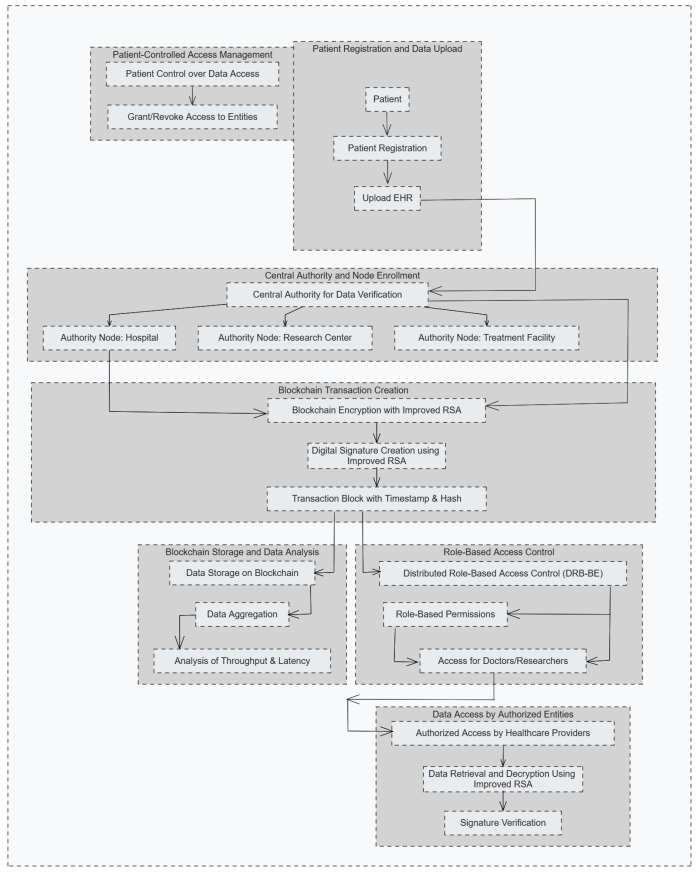
Proposed model.

This system architecture integrates cryptographic security, decentralized control access, and efficient management, which makes the Internet hospital governance service scalable and securely administered. The structured approach thus provides a robust solution for secure and efficient EHR management in blockchain-based Internet hospitals. An abstract view of the proposed methodology is provided in Figure 1.

**Figure 2:**
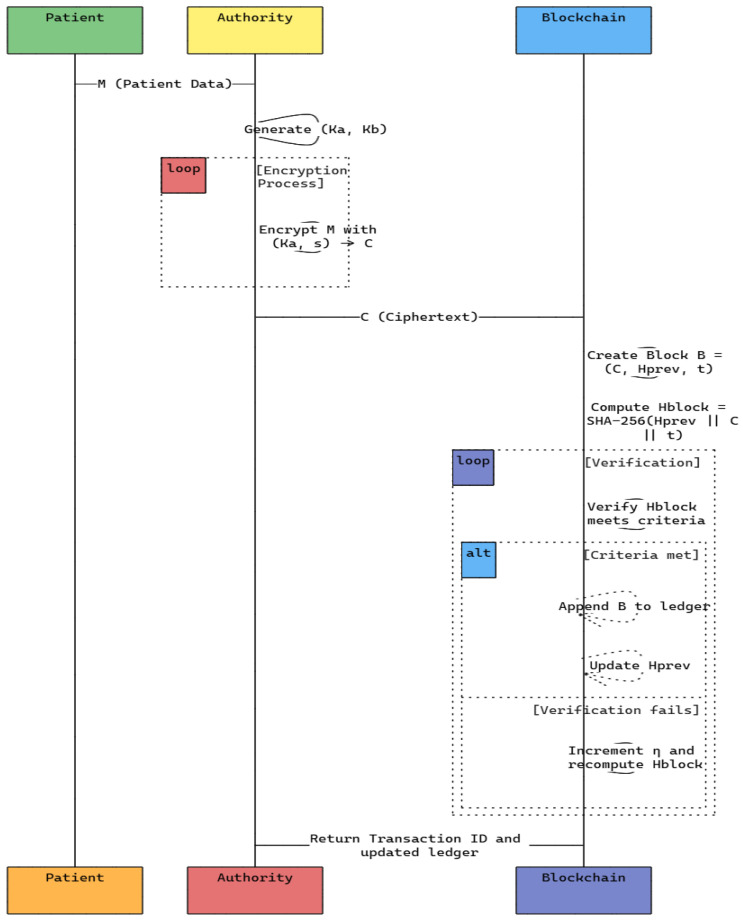
Sequence diagram of the proposed model.

Figure 2 illustrates the patient data registration and encryption in a blockchain-based healthcare system. The interaction begins with a patient submitting medical data to the authority for authentication. The authority requests to encrypt data using the advanced RSA scheme by encrypting the data of patients in a secure loop. The encrypted data are sent to a blockchain added to new transactions. A verification loop ensures that each block hash is correct and passes the integrity test. After successful verification, the transaction is added to the blockchain ledger. If the verification is unsuccessful, the hash is recalculated and continues running. It allows a strong base for storing patient records safely and gives a platform for handling data without centralization. Enhanced encryption and decentralized verification procedures are used.

Algorithm 1 defines three main steps: generating keys, message encryption, and decryption. This step first calculates two large primes, *p* and *q*, to make the modulus *n* using Euler’s Totient to obtain ϕ(n). The public key $K_{a}$ is computed randomly in [2,ϕ(n)] to become coprime with ϕn. The above modulus requires a scaling factor to change the scale, resulting in s=log(n)×1.15. The scaling factor is employed for the modulus size to fine-tune the modulus with improved security efficiency. The multiplication value 1.15 is a tune-in value applied for such applications that, for instance, to avoid an enormous overhead in medical health resource-constrained networks, require balancing the encryption strength over performance. Amplifying just a little to get log(n), the factor increases the complexity of the modulus arithmetic operations so that encryption would be hard against factorization attacks but must remain computationally tractable for real-time information processing. This factor was chosen based on empirical tests for achieving system robustness without overhead costs.
**Algorithm 1:** Improved RSA Key Generation, Encryption, and Decryption.**Input**: Two large primes p,q, Plaintext message *M***Output**: Ciphertext *C*, Decrypted message *M*  **1** **Step 1: Key Generation**;  **2** n←p×q;  **3** ϕ(n)←(p−1)(q−1);  **4** $K_{a}\leftarrow$ select random in range [2,ϕ(n)] such that $\gcd(K_{a},\phi \left(n\right))=1$;  **5** s←log(n)×1.15;  **6** $K_{b}\leftarrow K_{a}^{-1}\;\mathrm{mod}\;\phi \left(n\right)$;  **7** **Step 2: Encryption**;  **8** $C\leftarrow M^{K_{a}}\;\mathrm{mod}\;s$;  **9** **Step 3: Decryption**;**10** $M\leftarrow C^{K_{b}}\;\mathrm{mod}\;s$;**11** **return** C,M;

    Then, the private exponent $K_{b}$ can be generated using the modular inverse of $K_{a}$ modulo ϕ(n). For encryption, the public key $\left(K_{a},s\right)$ transforms plaintext *M* into ciphertext *C*. Finally, secret message *M* is recovered from ciphertext *C* using private key $\left(K_{b},s\right)$. The time complexity of the key-generation algorithm is $O(\log^{3}n)$, resulting from the computation of ϕ(n) and the modular inverse. In the encryption and decryption operations, the time complexity is $O(\log^{2}n)$ as it involves exponentiation modulo *s*. This trade-off shows that the algorithm can handle real-time applications and gives strong security against factorization attacks. This simple structure uses improved modulus to better security against factorization attacks without having slow operations on encryption and decryption.

Algorithm 2 represents the patient registration and transaction addition. It is a critical step that ensures data integrity and security on the blockchain. It enforces confidentiality, immutability, and traceability for the blockchain ledger. It is achieved by generating public and private keys using the RSA algorithm to encrypt the patient’s data. Ciphertext *C* is produced by encrypting patient data *M* through the help of public key $\left(K_{a},s\right)$. The patient data is encrypted using the improved RSA algorithm to form ciphertext *C*. The new block $B=(C,H_{\mathrm{prev}},t)$ is created, where $H_{\mathrm{prev}}$ represents the hash of the previous block and *t* is the timestamp. The hash of the block $H_{\mathrm{block}}$ is calculated using SHA-256, and a nonce η is incremented until the hash satisfies the verification conditions. Hashing has a computational complexity of O(k), where *k* is the length of the input data, while block verification has a complexity of O(m×k), where *m* is the number of increments of the nonce. This ensures data integrity and immutability with low processing overhead.

Algorithm 3 translates access management based on role-based permissions to DRB-BE. Subsequently, this will be mapped down to nodes that comprise a single authority, such as hospitals and research centers, plus its attributes, implementing attribute-based access control. The DRB-BE scheme enforces the role-based access control principle, granting access only to authorized users with predefined attributes related to patients’ data. This process begins by defining the access policy represented as P by mapping user roles and attributes that determine the right to access by doctors, nurses, and researchers. Then, the user’s attributes *U* are cross-checked against the needed ones *A*, as the access policy states. It gives the decryption right only if all attributes of a user are qualified with respect to requirements in a policy. An authorized user may then decrypt the encrypted data *C* with his private key, namely $\left(K_{b},s\right)$, to access his original message *M*. The complexity of attribute matching is O(|A|), where |A| is the number of attributes in the policy, while decryption complexity is still $O(\log^{2}n)$. This ensures efficient and secure access management in decentralized healthcare systems. Table 2 summarizes the time and space complexity of the proposed algorithms along with the key features.
**Algorithm 2:** Patient Registration and Transaction Addition.
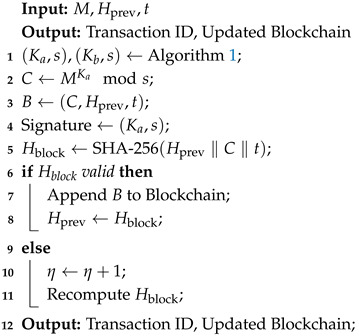


**Algorithm 3:** Role-Based Access Control with DRB-BE.

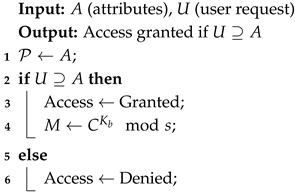



The validity of the blockchain is ensured by verifying the consistency of the hash for each block $B_{i}$, computed using Equation (1). Where H represents the hashing function, $I_{i}$ is the index of the block, $H(B_{i-1})$ is the hash of the previous block, $D_{i}$ is the data in the block, and $T_{i}$ is the timestamp. The validity is checked using the is_chain_valid method, which iterates through all blocks and verifies these conditions.

(1) $$H\left(B_{i}\right)=H(I_{i}||H\left(B_{i-1}\right)\left|\right|D_{i}\left|\right|T_{i})$$

The blockchain is valid if it satisfies Equation (2).

(2) $$\forall i\in \left[1,N\right],\;H\left(B_{i}\right)=H(I_{i}||H\left(B_{i-1}\right)\left|\right|D_{i}\left|\right|T_{i})$$

## 5. Experimentation and Results

This section presents the experimentation setup and a discussion of the results.

### 5.1. Experimentation Setup

This experimentation phase was planned in detail to test the proposed model’s performance by simulating an environment that closely resembles the Internet hospital application. The experiment involved hardware, software, and parameter configurations to be reliable and reproducible when considering multiple performance metrics against the baseline encryption methodology. The dataset includes EHRs for 1000 simulated patients, allowing robust encryption, decryption, and blockchain transaction handling testing.

The hardware used was based on a system running an Intel Core i7 processor (ThinkPad, Lenovo, Made in China) with eight cores clocked at 2.8 GHz, 32 GB RAM, and a 512 GB SSD. The setup included Google Colab Pro with the acceleration of GPU that helped speed up processes for cryptography and blockchain significantly more quickly. The experiment environment used Python 3.8 and libraries like pandas for data manipulation, NumPy for numerical computation, matplotlib and seaborn for visualization, hashlib for cryptographic hashing, time for execution time measurement, and sys for the memory usage analysis.

A structured parameter setup guided the experiment, both cryptographic and blockchain-specific configurations. The key parameters were 1024-bit RSA for encryption; SHA-256 hashing for authentication of the patient data being secured; dynamically generated keys $\left(K_{a},s\right)$ for every transaction; and computed private keys $\left(K_{b},s\right)$ using a modular inverse to decrypt each transaction. The blockchain utilized a block size of 1 KB, optimized and designed to manage transactions efficiently. In addition, the consensus mechanism is Proof of Work (PoW) to facilitate the secure validation of transactions through the network. The detailed parameter configurations are summarized in Table 3.

The experiment tested key performance metrics, including encryption and decryption time, transaction throughput, latency, and memory consumption. All these metrics give insight into how efficient and scalable the proposed model is compared with traditional methods. It allows for better computational speed, resource utilization, and transaction processing.

### 5.2. Results and Analysis

This section provides the results and comparisons of the proposed encryption model with the state of the art (i.e., ref. [31]; we refer to it as Modified RSA (Base) throughout this paper). Each metric is measured to determine the proposed model’s efficiency and scalability improvement compared with the baseline model. The proposed model exhibits many advancements over the baseline in multiple key performance metrics.

#### 5.2.1. Transaction Throughput

Transaction throughput, measured in Transactions Per Second (TPS), reflects the system’s capacity to handle a high volume of transactions simultaneously. The system’s throughput is defined as the number of transactions processed per second and computed using Equation (3), where *N* is the total number of transactions and $T_{\mathrm{latency}}\left(M_{i}\right)$ is the latency for the *i*-th transaction. This metric is computed in the code by dividing *N* by the total latency of all transactions.

(3) $$\mathrm{TPS}=\frac{N}{\sum_{i=1}^{N}T_{\mathrm{latency}}\left(M_{i}\right)}$$

**Figure 3:**
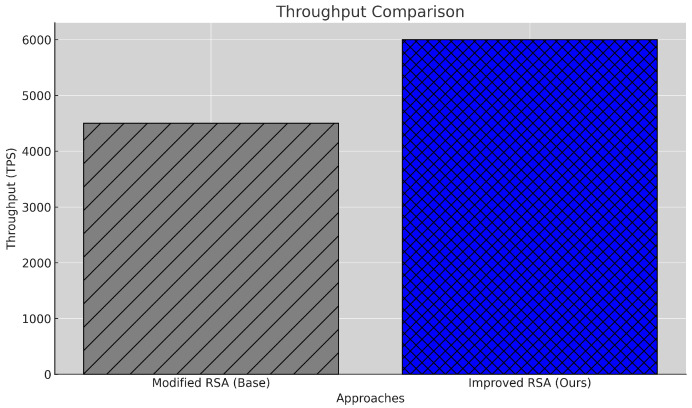
Transaction throughput comparison between baseline and proposed models.

Figure 3 demonstrates a significant improvement, with the proposed model achieving 5950 TPS compared to the baseline’s 4771 TPS, marking a 24.7% increase. This enhancement is primarily due to optimized data handling and block creation processes that reduce computational bottlenecks in the blockchain framework. The higher throughput is essential for real-time, data-intensive environments like Internet hospitals, where the timely processing of patient data is critical. The observed improvement underscores the scalability of our model, indicating that it can efficiently support an increasing volume of transactions as the healthcare network expands.

#### 5.2.2. Memory Usage

Efficient memory usage is crucial in resource-constrained environments, particularly healthcare IoT setups. The memory usage for storing encrypted messages across *N* transactions is computed using Equation (4). In the implementation, the Python sys.getsizeof function calculates the memory consumed by each ciphertext $C_{i}$. The total memory usage is obtained by summing the sizes of all *C*.

(4) $$\mathrm{Memory}\;\mathrm{Usage}=\sum_{i=1}^{N}\mathrm{sizeof}\left(C_{i}\right)$$

**Figure 4:**
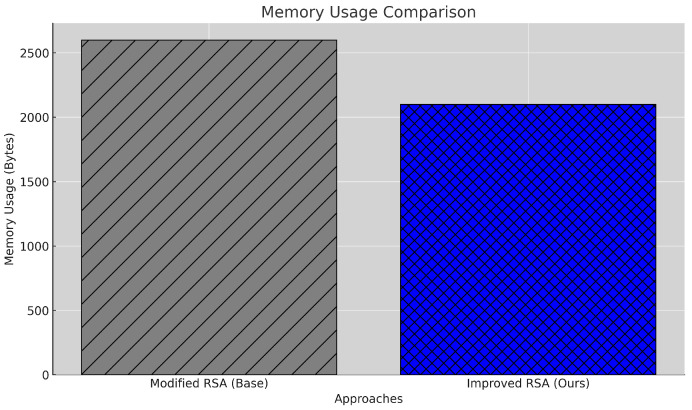
Memory usage comparison between baseline and proposed models.

As shown in Figure 4, the proposed model reduces memory usage by 14.3%, from 2800 bytes to 2400 bytes. This reduction is achieved through a streamlined encryption process and a more compact block structure, reducing data redundancy in the blockchain. By optimizing memory consumption, the model is well suited to deployment on devices with limited memory resources, such as those commonly used in healthcare IoT applications. However, while the reduction is beneficial, further optimization could reduce memory usage, enabling deployment on even more resource-limited devices.

#### 5.2.3. Latency

Latency, the delay between transaction initiation and addition to the blockchain, is critical in applications requiring real-time data processing, such as healthcare. The latency for a single transaction, including encryption and decryption, is presented in Equation (5).

(5) $$T_{\mathrm{latency}}\left(M_{i}\right)=T_{\mathrm{enc}}\left(M_{i}\right)+T_{\mathrm{dec}}\left(C_{i}\right)$$

The average latency across all *N* transactions is computed using Equation (6). The latency for each transaction is calculated by summing the encryption and decryption times stored in the corresponding lists. The average latency is obtained by dividing the total latency by *N*, the number of transactions.

(6) $${\bar{T}}_{\mathrm{latency}}=\frac{1}{N}\sum_{i=1}^{N}T_{\mathrm{latency}}\left(M_{i}\right)$$

**Figure 5:**
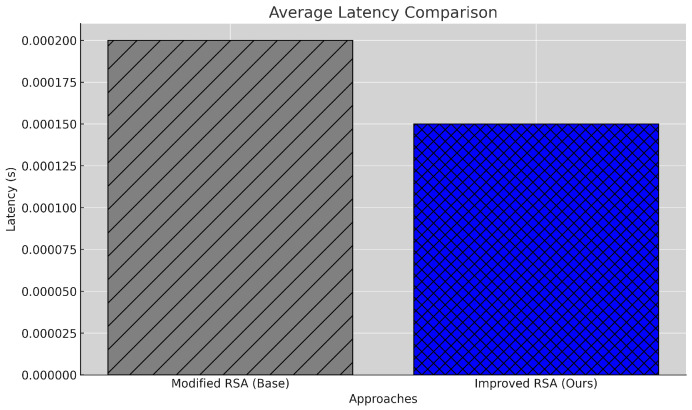
Latency comparison between baseline and proposed models.

The proposed model achieves an average latency of 0.000168 s, a 19.6% reduction from the baseline latency of 0.000209 s, as illustrated in Figure 5. This improvement is attributed to optimized block verification processes that reduce waiting times for transaction confirmation. The reduced latency enhances the system’s responsiveness, making it more suitable for time-sensitive applications in healthcare. However, while latency reduction is achieved, there remains room for further improvement, especially in scenarios where milliseconds of delay could impact critical decision making. Future research could explore lightweight consensus mechanisms or additional optimizations in the verification process to further reduce latency.

#### 5.2.4. Encryption Time

**Figure 6:**
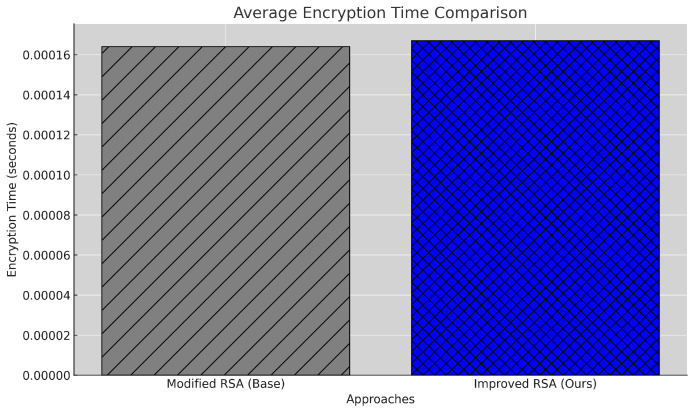
Encryption time comparison between baseline and proposed models.

Encryption time measures the time required to encrypt patient data and impacts overall system efficiency. The encryption time for a message $M_{i}$ is calculated using Equation 7 as the difference between the start and end times during encryption.

(7) $$T_{\mathrm{enc}}\left(M_{i}\right)=t_{\mathrm{end}}\left(E\left(M_{i},K\right)\right)-t_{\mathrm{start}}\left(E\left(M_{i},K\right)\right)$$

where $E(M_{i},K)$ represents the encryption of message $M_{i}$ using the public key *K*. It is computed using Python’s time module, where $t_{\mathrm{start}}$ and $t_{\mathrm{end}}$ capture the timestamps before and after the encryption process, respectively. The results for all messages are stored in the encryption_times_modified and encryption_times_improved lists. As seen in Figure 6, the proposed model shows a slight increase in encryption time, recording 0.000167 s compared to the baseline’s 0.000164 s, an increase of approximately 1.8%. This increase is due to additional security layers introduced in the encryption process to enhance data protection. Although this trade-off results in a minor delay, it remains in acceptable limits and is justified by the enhanced security provided by the proposed model. Nonetheless, this increase highlights a potential area for future improvement, where further optimization of the encryption algorithm could reduce processing time without compromising security.

#### 5.2.5. Decryption Time

Decryption time, representing the time taken to retrieve original data from ciphertext, is crucial for systems requiring rapid access to data. The decryption time for an encrypted message $C_{i}$ is computed using Equation (8), where $D(C_{i},K^{-1})$ is the decryption of $C_{i}$ using the private key $K^{-1}$. The decryption process timings are measured using the same approach as encryption and stored in the decryption_times_modified and decryption_times_improved lists.

(8) $$T_{\mathrm{dec}}\left(C_{i}\right)=t_{\mathrm{end}}\left(D\left(C_{i},K^{-1}\right)\right)-t_{\mathrm{start}}\left(D\left(C_{i},K^{-1}\right)\right)$$

**Figure 7:**
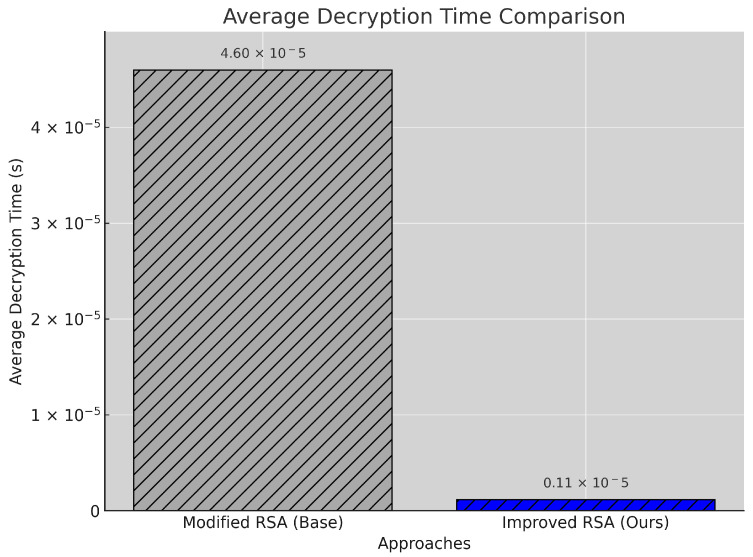
Decryption time comparison between baseline and proposed models.

Figure 7 shows a notable improvement in decryption speed, with the proposed model achieving an average decryption time of 1.15 × $10^{-6}$ s, a 97.5% reduction from the baseline’s 4.60 × $10^{-5}$ s. This substantial decrease in decryption time results from a streamlined decryption process that minimizes computational complexity, allowing faster data retrieval. This improvement is particularly significant for real-time healthcare applications where quick access to encrypted data can directly impact patient care. The significant reduction in decryption time underscores the proposed model’s suitability for scenarios where rapid data access is essential, such as in emergency or critical care settings.

#### 5.2.6. Statistical Validation of Performance Metrics

To validate the reliability of the performance metrics, statistical analyses were conducted on throughput and latency. The confidence intervals (CIs) for throughput and latency are computed using Equation (9), where $\bar{x}$ is the mean, $t_{\alpha /2,\nu }$ is the critical t-value, *s* is the sample standard deviation, and *n* is the sample size.

(9) $$\mathrm{CI}=\bar{x}\pm t_{\alpha /2,\nu }\frac{s}{\sqrt{n}}$$

**Figure 8:**
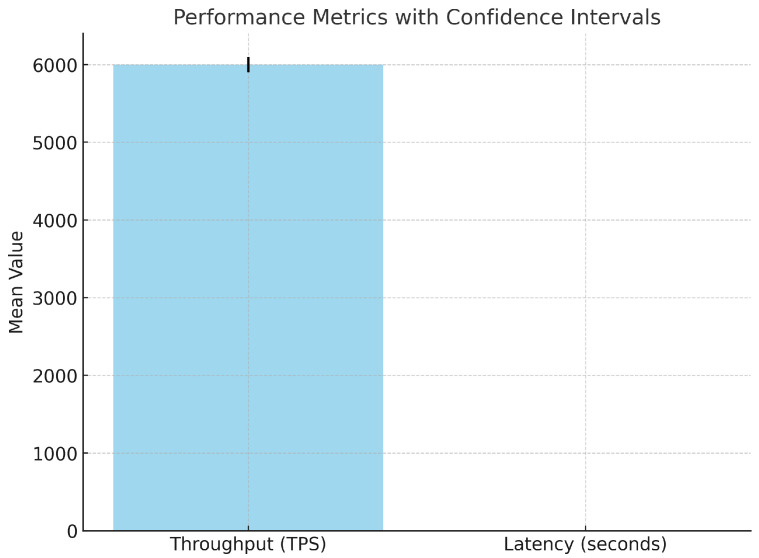
Performance metrics with confidence intervals.

Table 4 summarizes the statistical results for throughput and latency, including mean values, confidence intervals, and *p*-values. Figure 8 provides a graphical comparison with error bars representing the confidence intervals.

The mean throughput was 6000 TPS with a 95% confidence interval of [5901.84, 6098.16]; thus, reliable and consistent performance was realized. Latency was averaged to be 0.166 s with a 95% confidence interval of [0.1518, 0.1802], showing efficiency in low-latency operations by the model. Statistical validation further confirmed the importance of the throughput improvement as compared to the baseline model, with a *p*-value of $6.50\times 10^{-10}$. These results prove the proposed system to be robust and scalable, hence suitable for applications in real time like Internet hospitals.

#### 5.2.7. Discussion

The proposed blockchain-integrated encryption model presents a new approach to solving the security and performance challenges in healthcare data management, especially in Internet hospitals. It optimizes the RSA encryption process and embeds it into a blockchain framework. It provides a secure and scalable solution for the real-time handling of sensitive patient data. These are significant improvements in throughput, memory, latency, and decryption time. It also indicates the model’s capability of improving performance without sacrificing security. Analysis-wise, the proposed model is well suited to high-frequency transaction environments since it increases transaction throughput and reduces latency. The 24.7% increase in transaction throughput, combined with the 19.6% reduction in latency, means that optimized blockchain operations can handle the high volume of transactions characteristic of healthcare systems. Adding IoT sensors to the proposed system brings the critical dimension of allowing for real-time monitoring and management of patient data. The data streams generated from sensors are encrypted and stored on the blockchain, preserving privacy and integrity. For applications such as emergency healthcare and remote patient monitoring, real-time capability is important because patient outcomes are directly affected by fast and secure data processing.

In addition, reduced memory usage addresses the already low-resourced IoT devices in healthcare infrastructure. It also allows for more efficient use of system resources. These performance improvements indicate that the proposed model addresses the scalability and responsiveness of current healthcare systems. Although the proposed model has some substantial benefits, it also has certain limitations that must be acknowledged. Even though highly secured, the encryption process caused a slight increase in encryption time that may pose a potential threat in time-sensitive scenarios. This slight increase in size has no significant impact on performance. However, it suggests that other optimizations are required in resource-constrained environments. Given its security strength, the RSA encryption makes the algorithm less efficient. The chosen scheme may be less efficient than lightweight cryptographic schemes, making the code faster and consuming fewer resources. The dependence on IoT sensors would pose a potential threat related to failures or data inaccuracies, impacting the quality of blockchain transactions. Future work could be aimed at fault-tolerant mechanisms for addressing these issues.

With the security and data integrity guaranteed through the Proof-of-Work (PoW) consensus mechanism come extra computational overhead and energy consumption. However, with an emphasis on efficiency, resource constraints are considered favorable: such devices as devices at the edge would require less power and less computing capacity, making reliance upon them viable. Alternative mechanisms for building consensus will then be presented, which in the following section may involve alternative mechanisms and proof of stake or lighter protocols. Several future research directions will further enhance the proposed model. Advanced cryptography techniques such as homomorphic or attribute-based encryption are better layers of privacy and can be introduced. Such techniques will allow complex operations on data without revealing sensitive information. That would be of immense value in healthcare because the top priority of patient information is confidentiality. In addition, the real-life testing of the model in actual operational healthcare settings will be insightful regarding its applicability impact. It can be tweaked to address the operational issues that may have gone unconsidered or unnoticed. Such a deployment will also serve as an ideal check of the adaptability and reliability of the model in various healthcare settings.

To that extent, the interoperability model could be extended using current standards and systems in healthcare data, and it includes HL7 and FHIR to let data flow fluidly across different platforms and healthcare institutions. It will further improve usability as more comprehensive domains are important for sharing data in patient-centered healthcare systems. Further work could also look into the model’s scalability in larger-scale implementations, focusing on how a system would deal with huge data volumes and massive transactions over long periods. Although the presented model represents a promising, safe healthcare data management solution, limitations exist in different areas. Further refinements and compliance with the changing requirements in digital healthcare could be achieved by refining these limitations through advanced cryptography techniques, alternative consensus mechanisms, real-world testing, and enhanced interoperability. Future directions for blockchain healthcare systems reveal the prospect for further development and optimization so that these systems may ultimately be indispensable in the secure data management of patients in this digital era. Future work can be carried out on lightweight consensus mechanisms or edge-based processing to make the system highly optimizable on IoT sensor networks, especially in areas with resource constraints.

## 6. Conclusions and Future Work

The increasing demand for Internet hospitals and digitization in health services demands robust data management and security. The conventional encryption models ensure satisfactory data protection while having some performance degradation. With unique inherent characteristics like transparency, immutability, and security, blockchain handles the security requirements of data management. The significant issue in integrating blockchain and encryption is the lack of satisfaction demanded by real-time healthcare systems. It causes high latency, low throughput, and resource-constrained networks. To reduce such limitations, this paper proposed an improved model integrating private blockchain into encryption. This model offers a balanced approach to satisfying Internet hospitals’ security and performance requirements. It optimizes the RSA encryption process and incorporates it into a blockchain framework. This model has enhanced data security while maintaining scalability and responsiveness for dealing with sensitive patient information in real time. It further enriches the blockchain-based health system, which is structured and efficient in securing data management in Internet hospitals by integrating blockchain technology into optimized encryption schemes. Therefore, all these limitations could be overcome and pave the way toward more effective and reliable systems in health data. This, in turn, enhances data privacy and access control while aligning with the performance and scalability requirements needed in the healthcare industry. The system realizes real-time data acquisition and secure processing, giving rise to the critical need for patient monitoring and remote healthcare services. Sensor data are integrated into the blockchain to ensure privacy and immutability. The system is a robust solution for health-related data management in the Internet hospital setup. The proposed alliance blockchain model significantly improved the performance of Internet hospitals. The throughput was improved by 24.7% and latency was reduced by 19.6% compared to the baseline. Statistical validation of the result showed a throughput with a 95% confidence interval and a *p*-value of $6.50\times 10^{-10}$, confirming the robustness and reliability of the proposed model. Such advancements showcase the system’s capability of handling real-time healthcare data securely and efficiently while ensuring scalability and responsiveness for critical applications. Further work in this area could include improving cryptographic techniques that would increase data privacy with additional layers of security and efficiency in cryptographic techniques, such as homomorphic or attribute-based encryption.

## Figures and Tables

**Table 1:** Comparison of state of the art.

Ref.	Methodology	Advantages	Limitations
[26]	Blockchain with digital signatures.	Collusion resistance, data integrity.	High computational cost.
[27]	ABE with non-monotone predicates.	Flexible access control.	Attribute verification overhead.
[28]	Multi-authority ABE.	Prevents forgery, preserves privacy.	Complex implementation.
[29]	Federated health alliance with blockchain.	Secure data sharing.	High latency.
[30]	Blockchain + IPFS storage.	Efficient storage and retrieval.	Poor scalability.
[31]	RSA with SHA-256 in blockchain.	Strong attack defense.	Computational overhead.
[32]	ABE with role-based access.	Enhanced confidentiality.	Resource constraints.
[39]	Blockchain + IPFS with DNA encoding and AES.	Multilayer security.	High computational cost.
[40]	RSA-encrypted NFTs and smart contracts.	Transparent pediatric record management.	RSA/NFT complexity.
[41]	RSA-encrypted NFTs for pet health.	Secure data management.	NFT implementation complexity.

**Table 2:** Complexity analysis of Algorithms 1–3.

Algorithm	Time Complexity	Space Complexity	Key Features
Improved RSA (KeyGen, Encrypt, Decrypt)	$O\left(\log^{3}n\right),O\left(\log^{2}n\right)$	O(n)	Optimized decryption, secure encryption
Patient Registration and Transaction	O(k),O(m×k)	O(k)	Data immutability, secure transaction storage
Role-Based Access Control (DRB-BE)	$O\left(|A|\right),O\left(\log^{2}n\right)$	O(|A|)	Decentralized and secure access management

**Table 3:** Experimental parameters for encryption and blockchain setup.

Parameter	Value	Description
Number of Patients	1000	Total records processed
RSA Key Length	1024 bits	Key length for RSA encryption
Encryption Algorithm	SHA-256	Algorithm for hashing patient data
Public Key $\left(K_{a},s\right)$	Generated per transaction	Public key based on selected primes
Private Key $\left(K_{b},s\right)$	Computed using modular inverse	Key used for decryption
Block Size	1 KB	Maximum block data size in blockchain
Blockchain Consensus	Proof of Work	Mechanism for transaction validation

**Table 4:** Statistical validation of performance metrics.

Metric	Mean Value	95% Confidence Interval	*p*-Value
Throughput (TPS)	6000.0	[5901.84, 6098.16]	$6.50\times 10^{-10}$
Latency (seconds)	0.166	[0.1518, 0.1802]	-

## Data Availability

Data are contained within the article.

## Abbreviations and Symbols

The following abbreviations and symbols are used in this manuscript:

**Abbreviations**	**Description**
DRB-BE	Distributed Role-Based Blockchain Encryption
RSA	Rivest–Shamir–Adleman
PoW	Proof of Work
TPS	Transactions Per Second
EHR	Electronic Health Record
IoT	Internet of Things
HL7	Health Level Seven
FHIR	Fast Healthcare Interoperability Resources
GPU	Graphics Processing Unit
SHA-256	Secure Hash Algorithm 256
PBFT	Practical Byzantine Fault Tolerance
FL	Federated Learning
CIs	Confidence Intervals
**Symbols**	**Description**
*M*	Patient Data (Plaintext Message)
*C*	Encrypted Data (Ciphertext)
$K_{a}$	Public Key (Encryption Exponent)
$K_{b}$	Private Key (Decryption Exponent)
p,q	Large Prime Numbers
*n*	RSA Modulus (n=p×q)
ϕ(n)	Euler’s Totient Function
*s*	Modified Modulus (s=log(n)×1.15)
$H_{\mathrm{prev}}$	Hash of the Previous Block
$H_{\mathrm{block}}$	Hash of the Current Block
*t*	Timestamp of the Transaction
η	Nonce used in Block Verification
P	Access Policy for Role-Based Access
*U*	User Attributes
*A*	Required Attributes
gcd	Greatest Common Divisor
log	Natural Logarithm
${\bar{T}}_{\mathrm{latency}}$	Average Latency of Transactions
CI	Confidence Interval
$t_{\alpha /2,\nu }$	Critical Value for Confidence Interval
*s*	Sample Standard Deviation
*n*	Sample Size
$\bar{x}$	Mean Value of Observed Data
$T_{\mathrm{enc}}\left(M_{i}\right)$	Encryption Time for Message $M_{i}$
$T_{\mathrm{dec}}\left(C_{i}\right)$	Decryption Time for Ciphertext $C_{i}$
$T_{\mathrm{latency}}\left(M_{i}\right)$	Total Latency for Message $M_{i}$
